# Low-dose post-transplant cyclophosphamide and anti-thymocyte globulin as an effective strategy for GVHD prevention in haploidentical patients

**DOI:** 10.1186/s13045-019-0781-y

**Published:** 2019-09-03

**Authors:** Yu Wang, De-Pei Wu, Qi-Fa Liu, Lan-Ping Xu, Kai-Yan Liu, Xiao-Hui Zhang, Wen-Jing Yu, Yang Xu, Fen Huang, Xiao-Jun Huang

**Affiliations:** 1 National Clinical Research Center for Hematologic Disease, Beijing Key Laboratory of Hematopoietic Stem Cell Transplantation, Peking University People’s Hospital, Peking University Institute of Hematology, No.11, Xizhimen South Street, Xicheng District, Beijing, 100044 China; 2 The first affiliated hospital of Soochow University, Suzhou, 215006 Jiangsu China; 3 Nanfang Hospital, Southern Medical University, Guangzhou, 510515 Guangdong China; 4 Peking-Tsinghua Center for Life Sciences, Beijing, 100871 China

**Keywords:** Anti-thymocyte globulin, Post-transplant cyclophosphamide, Low-dose, Haploidentical, Graft-versus-host disease

## Abstract

**Background:**

Low-dose post-transplant cyclophosphamide (PTCy) in conjunction with anti-thymocyte globulin (ATG) appears as a potentially effective graft-versus-host disease (GVHD) prevention strategy in haploidentical hematopoietic cell transplant (haplo-HCT). Our study aims to assess the efficacy of this regimen.

**Methods:**

We extended our prospective study in patients treated with low-dose PTCy (14.5 mg/kg on days 3 and 4) in ATG/granulocyte colony-stimulating factor (G-CSF)-based regimen and compared the results to the contemporary cohort of patients without low-dose PTCy (ATG cohort). Both study cohort and control are transplanted from maternal donor or collateral relatives.

**Results:**

We identified 239 consecutive patients (ATG-PTCy cohort = 114; ATG cohort = 125). All patients but one in ATG cohort achieved myeloid engraftment by day 30 post-HCT. We found that both the cumulative incidence of 100-day grade III–IV aGvHD and non-relapse-mortality (NRM) in the ATG-PTCy cohort was significantly reduced than that in the ATG group (5% vs 18%; *P* = 0.003; and 6% vs 15%; *P*= 0.045); the 2-year cumulative incidences of relapse and overall survival were comparable between the two cohorts (13% vs 14%; *P* = 0.62; and 83% vs 77%; *P* = 0.18, respectively). Furthermore, GVHD-free, relapse-free survival (GRFS) was significantly improved in the ATG-PTCy arm (63% vs 48%; *P* = 0.039). In multivariate analysis, the joint treatment resulted in lower grade II–IV acute GVHD (HR 0.58; *P* = 0.036), grade III–IV aGvHD (HR 0.28; *P* = 0.006), chronic GVHD (HR 0.60; *P* = 0.047), NRM (HR 0.26; *P* = 0.014), and higher GRFS (HR 0.59; *P* = 0.021) but slower myeloid and platelet recovery (HR 0.29 and 0.30; both *P* < 0.001).

**Conclusions:**

These results suggested that ATG/PTCy (low-dose) can reduce both acute and chronic GVHD as compared with standard ATG-based prophylaxis using maternal donor or collateral relatives at particular high GVHD risk.

## Background

During the past 20 years, wider application of easily available haploidentical donor hematopoietic cell transplant (haplo-HCT) has been made possible through the T cell-replete (TCR) regimens including T cell regulation with anti-thymocyte globulin (ATG)/granulocyte colony-stimulating factor (GCSF) and post-transplant cyclophosphamide (PTCy) [1–9]. To achieve decreased non-relapse mortality (NRM) and improved long-term outcomes in haploidentical transplant, the joint use of ATG and PTCy might effectively reduce graft-versus-host disease (GVHD) and mortality associated with severe forms of GVHD [10–14]. There are multiple papers using ATG/PTCy (high-dose) in patients with Fanconi anemia, aplastic anemia, and sickle cell disease [10]. To date, the combination of conditioning regimens with low-dose ATG and high-dose PTCy after haplo-HCT [11–14] or unrelated donor (URD) HCT [15, 16] for hematological malignancies has been documented in several reports with reduced rates of GVHD and acceptable relapse rate albeit with somewhat high rates of graft failure (8%) [11] or delayed engraftment [12]. Recently, results of a prospective trial have been available which analyze the efficacy of combined use of low-dose ATG and 1-day high-dose PTCy (50 mg/kg at + 3 days) in preventing GVHD among haplo patients [17].

Recently, we established a regimen using low-dose PTCy in conjunction with standard-dose ATG in order to lower the risk of GVHD without compromising engraftment and disease relapse. This protocol consisted of a myeloablative conditioning (MAC) regimen containing cytarabine, busulfan, Cy combined with standard-dose ATG/G-CSF (the so-called Beijing protocol), and followed by low-dose PTCy (14.5 mg/kg on days 3 and 4 after HCT) for haplo-HCT recipients. We previously reported this strategy in preliminary prospective study wherein reduced incidences of both acute and chronic GVHD were observed in comparison with non-combined regimens [18]. In this prospective trial, results indicated that low-dose PTCy is sufficient to lower acute GVHD in mouse model, partly due to the boosting of fast regulatory T cell (Treg) reconstitution. In addition, low-dose PTCy could augment the protective effect of ATG on GVHD both in mouse and human sets while ensuring high rates of engraftment and keeping disease control [18]. These results seem to highlight the feasibility of this novel procedure with low-dose PTCy in ATG-based MAC for GVHD prevention and perfect outcomes post-haplo-HCT. However, the potentially effective regimen needs to be validated on a larger population with hematological neoplasms.

With the aim to assess the efficacy of this regimen in haplo-HCT, we extended our prospective study in consecutive patients treated with ATG/GCSF-PTCy (low-dose) regimen and initiated a multicenter analysis on comparing the results to the contemporary cohort of patients who received ATG/GCSF without low-dose PTCy. In the current study, patients in both study group and contemporary control group are all transplanted from maternal donor (mother donors, MDs) [3] or collateral relative donors (CRDs, e.g., uncle, aunt, nephew, niece, and cousins) [19, 20] who had especially high risk of GVHD occurrence under “Beijing protocol.” Herein, we tested the efficacy of ATG/PTCy (low-dose) as compared with standard ATG-based prophylaxis using maternal donor or collateral relatives. Thus, this new approach in unmanipulated haplo-HCT with low-dose PTCy in ATG/GCSF-based MAC would expand the donor pool for patients eligible for allogeneic HCT, by allowing the safe and effective use of specific haploidentical donors associated with especially high risk of GVHD occurrence.

## Patients and methods

### Inclusion and exclusion criteria

Eligibility criteria included consecutive patients with hematological neoplasms, who underwent a first allo-HCT from April 2015 (start time of the clinical trial) and extended to June 2018, using either MDs or CRDs. Signed informed consent was given to all patients and donors. The study protocol was approved by the Institutional Review Board of each center and was in accordance with the Declaration of Helsinki. Patients were defined as having low-, intermediate-, or high-risk disease stages according to the established disease risk index [21, 22]. Exclusion criteria were contraindications to HCT such as severe heart, kidney, or liver function failure and a prior transplant.

### Donors

Donors are either MDs or CRDs. For haplo-HCT, young, male siblings or paternal donor is the first choice whereas MD or CRD is the last choice [3, 19, 23]. HLA typing was undertaken according to our previous protocol [24, 25].

### Transplant procedures

The conditioning regimen is a standardized ATG/GCSF-based MAC protocol (the so-called Beijing protocol) comprising of the following: cytarabine, 4 g/m^2^/day intravenously (i.v.) on days − 10 and − 9; busulfan (BU), 9.6 mg/kg i.v., given in 12 doses on days − 8 to − 6; Cy, 1.8 g/m^2^/day i.v. on days − 5 and − 4; simustine, 250 mg/m^2^, orally, on day − 3; and rabbit ATG (Sangstat-Genzyme) 2.5 mg/kg/day i.v., on days from − 5 to − 2. Two doses of 14.5 mg/kg Cy was given on days 3 and 4 post-HCT in ATG-PTCy cohort according to the trial protocol. Allografts were harvested and infused as previously described [24]. All patients received cyclosporine (CSA), mycophenolate mofetil (MMF), and methotrexate (MTX) for GVHD prophylaxis as detailed in previous report [26]. Methylprednisolone at 1 mg/kg/day i.v was given to patients developing grade II or greater acute GVHD (aGVHD). Steroid refractory aGVHD (SR aGVHD) was defined as progression of GVHD severity after 3 days of steroid therapy or no improvement after 5 days of steroid therapy. Patients with SR aGVHD received second-line treatment with basiliximab (Novartis Pharma AG, Basel, Switzerland) [26]. Details of cytomegalovirus (CMV) and Epstein-Barr virus (EBV) monitoring were described previously [24].

We used two methods for minimal residual disease (MRD) measurement in bone marrow samples at the time of transplant: (1) aberrant leukemia-associated immune phenotypes (LAIPs) detected by four-color flow cytometry (FCM) and (2) WT1 mRNA levels detected by polymerase chain reaction (PCR). FCM-positive was defined as > 0.01% of cells with a LAIPs phenotype in ≥ 1 bone marrow samples. WT1-positive was defined as a transcript level > 0.60% in ≥ 1 bone marrow samples. Subjects were defined as MRD positive if they were both FCM and WT1 positive in a single sample [22]. The chimerism of leukocytes was analyzed post-transplant utilizing polymerase chain reaction amplification of short tandem repeats (PCR-STR). Semi-quantitative detection of PCR-STR products was analyzed using denaturing high-performance liquid chromatography (DHPLC) at Peking University before year 2017, and afterward, capillary electrophoresis on ABI PRISM 3130 Genetic Analyzer was used for quantitative detection of PCR-STR while the other two centers consistently adopted the latter one.

## Definitions and statistical analysis

### Study design

The current study is an extension of our prospective study in which patients received ATG/GCSF-based MAC followed by low-dose PTCy (ATG-PTCy cohort, cohort A) at the Peking University People’s Hospital, according to the protocol of clinical trial (registered at http://clinicaltrials.gov/ NCT02412423). Results were compared with a contemporary external control cohort of patients also transplanted from MDs or CRDs who received the identical standardized ATG/GCSF-based MAC protocol (the so-called Beijing protocol, below described in detail), however without the addition of low-dose PTCy (ATG cohort, cohort B) at the first affiliated hospital of Soochow University or Nanfang Hospital, Southern Medical University. The current contemporary external control is distinguished from that in our previous report in which patients transplanted from donors other than MDs or CRDs without low-dose PTCy were analyzed as contemporary control. As all patients transplanted from MDs or CRDs received the combined treatment since the trial started and from then on at Peking University People’s Hospital, the other two most experienced transplant centers in China were chosen as external control. The transplant patient number in the three transplant centers ranked top 3 among public hospitals in Chinese Bone Marrow Transplantation Registry (CBMTR). Data for all the patients from the two cohorts are routinely prospectively collected using a predefined case report form.

### End points

The primary study end point was the incidence of acute GVHD, grades III–IV. Secondary end points were the engraftment rate, chimerism, the incidences of acute GVHD grades II–IV, infection, chronic GVHD, the cumulative incidences of NRM and relapse, and probability of disease-free survival (DFS), overall survival (OS), and GVHD-free, relapse-free survival (GRFS).

Definitions of myeloid and platelet recovery, engraftment, graft failure, infection, NRM, relapse, DFS, and OS were detailed previously [25]. Full donor chimerism was defined as ≥ 95% leukocytes of donor origin in peripheral blood samples [25]. GVHD was defined and graded according to published criteria [27, 28]. Adjudication of GVHD diagnosis used case report forms containing data regarding organ involvement, which were verified by medical record inspection. A panel of five blinded experts determined the presence and grade of GVHD based on these data. GRFS events were defined as grade III–IV aGVHD, chronic GVHD (cGVHD) requiring systemic immunosuppressive treatment, leukemia relapse, or death from any cause during follow-up after allo-HSCT

### Statistical analyses

Groups were compared with the *χ*^2^ statistic for categorical variables and the Mann–Whitney test for continuous variables. Competing risk model was used to calculate cumulative incidences, with relapse treated as a competing event for NRM and with death from any cause as a competing risk for engraftment, GVHD, CMV, or EBV reactivation, and relapse. The Kaplan–Meier curves were used to estimate DFS, OS, and GRFS probabilities. Variables in Table 1 were included into a Cox proportional hazard model. Alpha was set at 0.05. All *P* values are two-sided. SAS V. 9.3 (SAS Institute Inc., Cary, North Carolina, USA) and SPSS 19.0 (Mathsoft, Seattle, WA, USA) were used for data analyses.

**Table 1:** Subject-, disease-, and transplant-related variables

Characteristics	ATG-PTCy cohort (*n* = 114)	ATG cohort (*n* = 125)	*P* value
Recipient age (year, median, range)	27 (5–52)	24 (4–65)	0.004
HLA-matching (A, B, DR)			0.001
3/6	94 (82%)	78 (62%)	
4/6 or 5/6	20 (18%)	47 (38%)	
Recipient gender			0.35
Male	63 (55%)	77 (62%)	
Female	51 (45%)	48 (38%)	
Disease type			0.99
Acute myeloid leukemia (AML)	55 (48%)	59 (47%)	
Acute lymphoblastic leukemia (ALL)	47 (41%)	52 (42%)	
Myelodysplastic syndromes (MDS)	9 (8%)	11 (9%)	
Chronic myeloid leukemia (CML)	3 (3%)	3 (2%)	
Disease risk index			0.20
Low/intermediate	101 (88%)	103 (83%)	
High/very high	13 (12%)	22 (17%)	
Minimal residual disease at transplant			0.86
Negative	93 (82%)	104 (83%)	
Positive	21 (18%)	21 (17%)	
Donor source			0.19
Mother	80 (70%)	98 (78%)	
Collateral relatives	34 (30%)	27 (22%)	
Donor gender male vs female	27 vs 7	20 vs 7	
Donor age (year, median, range)	46 (12–67)	42 (10–61)	
Maternal vs paternal line	20 vs 14	19 vs 8	
Donor gender			0.15
Male	27 (24%)	20 (16%)	
Female	87 (76%)	105 (84%)	
Donor-recipient pair			0.20
Female to male	68 (60%)	64 (51%)	
Others	46 (40%)	61 (49%)	
Donor age (year, median, range)	46 (12–67)	42 (10–61)	0.002
Blood type matching			0.020
Match	51 (45%)	75 (60%)	
Mismatch	63 (55%)	50 (40%)	
Median mononuclear cell (range, 10^8^/kg)	8.2 (3.3–11.8)	10.4 (3.8–26.1)	< 0.001
Median CD34^+^ cells (range, 10^6^/kg)	1.9 (0.5–14.1))	3.4 (1.6–21.2)	< 0.001
Median CD3^+^ cells (range, 10^8^/kg)	1.4 (0.3–5.7)	1.5 (0.3–11.1)	0.63
Year of transplant			0.006
April 2015 to August 2016	40 (35%)	66 (53%)	
September 2016 to June 2018	74 (65%)	59 (47%)	
Median follow-up (range, days)	587 (57–1399)	682 (3–1448)	0.20
Median follow-up in survivors (range, days)	661 (128–1399)	859 (230–1448)	0.007

## Results

### Study population

A total of 239 consecutive patients (ATG-PTCy cohort = 114, cohort A; ATG cohort = 125, cohort B) were identified eligible for this study (maternal donors = 178; collateral relatives = 61). Forty patients in ATG-PTCy cohort transplanted before August 2016 [18] was previously reported and further followed in the current study.

Patient and donor characteristics are shown in Table 1. No significant difference was observed between groups in terms of patients’ gender, gender mismatch, disease type, and disease risk index (DRI). However, patients in cohort A had higher percent of 3/6 HLA-A, B, and DR matching and lower percent of ABO blood type matching with donor as well as lower number of infused mononuclear cells (MNC) as compared to those in cohort B. Patients or donors in cohort B were 3 and 4 years younger than that in cohort A, respectively. The detailed information on the “collateral relatives” was shown in Table 1.

### Low-dose PTCy significantly reduced acute and chronic GvHD

The 100-day cumulative incidence of grade III–IV aGVHD in cohort A with low-dose PTCy was significantly reduced as compared to that in cohort B without low-dose PTCy (5% vs 18%; *P* = 0.003) and the rate for grade II–IV aGVHD in cohort A had a trend to be lower than that in cohort B (26% vs 36%; *P* = 0.14).

The 2-year cumulative overall incidences of chronic GVHD in cohort A with low-dose PTCy had a trend to be decreased in comparison with that in cohort B without low-dose PTCy (30% vs 44%; *P* = 0.07). However, the rate of moderate-to-severe chronic GVHD in cohort A was comparable to the rate in cohort B (17% vs 16%; *P* = 0.71).

As shown in Table 2, the use of low-dose PTCy was a significantly protective factor for grade II–IV aGVHD and grade III–IV aGvHD as well as chronic GVHD in multivariate analysis.

**Table 2:** Multivariate analyses of outcomes

Outcome	Hazard ratio (95% CI)	*P* value
ANC recovery		
Cohort ATG-PTCy vs ATG	0.29 (0.21–0.41)	< 0.001
Other significant factors		
Mononuclear cell count	1.09 (1.04–1.14)	< 0.001
CD34^+^ cell count	1.08 (1.01–1.15)	0.012
Platelet recovery		
Cohort ATG-PTCy vs ATG	0.30 (0.21–0.42)	< 0.001
Other significant factors		
Mononuclear cell count	1.06 (1.01–1.11)	0.008
Acute GvHD ≥ grade 2		
Cohort ATG-PTCy vs ATG	0.58 (0.35–0.96)	0.036
Acute GvHD ≥ grade 3		
Cohort ATG-PTCy vs ATG	0.28 (0.11–0.69)	0.006
Total chronic GvHD		
Cohort ATG-PTCy vs ATG	0.60 (0.38–0.99)	0.047
Non-relapse mortality		
Cohort ATG-PTCy vs ATG	0.26 (0.08–0.75)	0.014
Disease risk index		
Low/int vs high	0.28 (0.12–0.68)	0.005
Relapse		
Cohort ATG-PTCy vs ATG	0.65 (0.28–1.50)	0.31
Disease-free survival		
Cohort ATG-PTCy vs ATG	0.44 (0.22–0.86)	0.016
Disease risk index		
Low/int vs high	0.51 (0.27–0.99)	0.048
Overall survival		
Cohort ATG-PTCy vs ATG	0.47 (0.22–1.01)	0.055
Disease risk index		
Low/int vs high	0.48 (0.23–0.99)	0.048
GVHD and relapse-free survival		
Cohort ATG-PTCy vs ATG	0.59 (0.38–0.92)	0.021

### Hematopoietic recovery, infection, and transplant outcomes

All patients achieved myeloid engraftment by day 30 post-HCT except one in the ATG cohort who died of infection at day 11 post-HCT without myeloid recovery. The median value of donor chimerism at day 30 post-HCT was 100% (range, 95–100%) in cohort A and 97.3% (range, 73.9–100%) in cohort B. The median time to myeloid recovery was 3 days shorter in cohort B (12 days, range, 10–17 days) than in cohort A (15 days, range, 7–23 days; *P* < 0.001). The 100-day platelet recovery was significantly lower in cohort A than that in cohort B (90% vs 97%; *P* = 0.003). A multivariate analysis indicated that the use of low-dose PTCy significantly delayed myeloid and platelet recovery (Table 2).

The 100-day cumulative incidence of CMV reactivation in ATG-PTCy cohort was significantly higher than that in ATG cohort (74% vs 30%; *P* < 0.001) while the 100-day cumulative incidence of CMV disease was comparable between the two cohorts (8% vs 8%; *P* = 0.95). The 100-day cumulative incidence of EBV reactivation and post-transplant lympho-proliferative disorder (PTLD) was comparable (21% vs 20%; *P*=0.83; and 3% vs 2%; *P* = 0.57; respectively).

Up to Jan 31, 2019, the median follow-up time among survivors in cohorts A and B was 661 days (range, 128–1399) and 859 days (range, 230–1448) post-HCT, respectively (*P* = 0.007, Table 1). The 2-year cumulative incidences of relapse were similar among the two cohorts (13% vs 14% for cohort A vs cohort B; *P* = 0.62), and the relapse rate was 15% and 9% (*P* = 0.61) in groups A vs B in the subgroup analysis of high-risk disease. The 2-year cumulative incidence of NRM in study cohort A with low-dose PTCy was significantly reduced as compared with that in cohort B (6% vs 15%; *P* = 0.045). The 2-year probability of DFS had only a trend to be higher in cohort A than that in cohort B (81% vs 71%; *P* = 0.06) while OS was comparable between the two cohorts (83% vs 77%; *P* = 0.18). However, the 2-year probability of GRFS in study cohort A with low-dose PTCy was significantly improved as compared with that in cohort B (63% vs 48%; *P* = 0.020). As shown in Table 2, the use of low-dose PTCy significantly decreased NRM as well as increased DFS and GRFS in cohort A as compared with that in cohort B in the multivariate analysis.

**Fig. 1:**
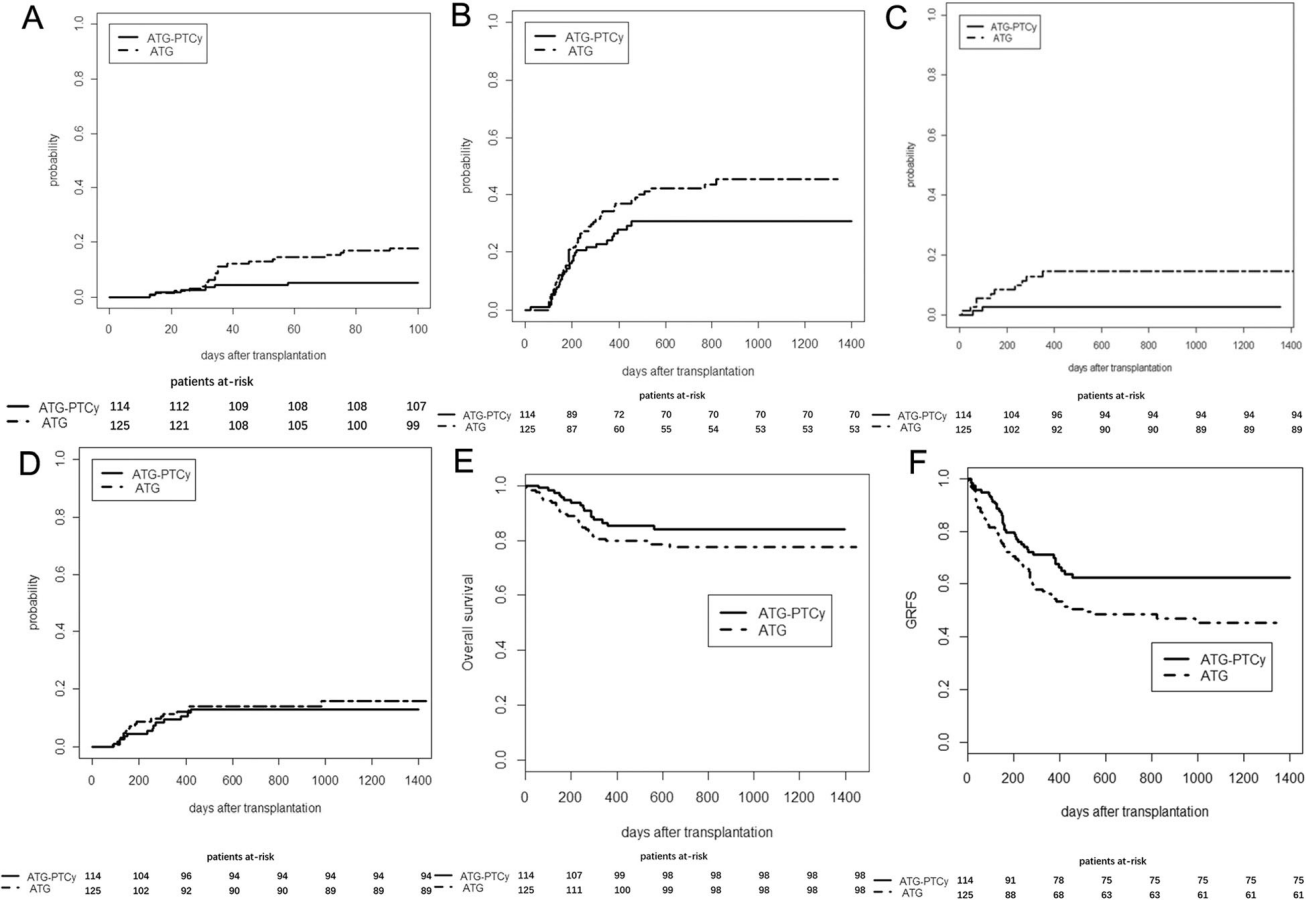
Rates of graft-versus-host disease (GVHD), non-relapse-mortality (NRM), relapse incidence (RI), overall survival (OS), and GVHD-free, relapse-free survival (GRFS) according to treatment group. Rates of grades III–IV acute GVHD (**a**), chronic GVHD (**b**), NRM (**c**), RI (**d**), OS (**e**), and GRFS (**f**)

Grade III–IV aGvHD (*n* = 3) or severe cGVHD (*n* = 4) accounted for 26% of death in the ATG cohort as compared to none in the ATG-PTCy cohort while disease relapse was the most common cause of death (56%) in the ATG-PTCy cohort (Table 3). Incidences of GVHD, NRM, and relapse as well as probability of OS and GRFS are shown in Fig. 1.

**Table 3:** Causes of death

Cause of death	ATG-PTCy cohort (*n* = 16)	ATG cohort (*n* = 27)
Relapse	9 (56%)	8 (30%)
GVHD	0	7(26%)
Infection other than CMV/EBV	5 (31%)	10 (37%)
CMV disease	1 (6%)	0
Organ failure	1 (6%)	2 (7%)

## Discussion

We compared our novel GVHD prevention strategy (low-dose PTCy in ATG/GCSF-based regimen) with contemporary control patients all transplanted from maternal donors or collateral relatives at particular high GVHD risk [3, 19, 20]. Cumulative incidence of acute GVHD, grade III–IV aGVHD, and chronic GVHD was significantly lower without GVHD-related death in the ATG-PTCy cohort as compared with the control cohort. Reliable engraftment and disease control have also been achieved. As a result, significantly decreased NRM and improved GRFS have been accomplished. Our observations reveal that low-dose PTCy combined with ATG/GCSF-represented conditioning could be a promising new regimen for GVHD prophylaxis and improve outcomes after haplo-HCT.

The current results suggest that the administration of low-dose PTCy along with ATG/GCSF significantly decreased the rate of both acute and chronic GVHD as compared to contemporary controls who were also transplanted from MDs or CRDs proved to be at particular high GVHD risk under “Beijing protocol” in both single-center and multi-center studies [3, 5, 6, 19, 20]. Furthermore, grade III–IV aGvHD or severe cGVHD GVHD was the main cause of death in 7 out of 27 patients in the control cohort, in contrast to none in the combined treatment cohort (Table 3). It should be stated that despite the significantly decreased rate of grade III–IV aGvHD and total chronic GVHD in the ATG-PTCy cohort compared to control cohort, the incidence for moderate to severe chronic GVHD remains higher. And the rate of moderate to severe chronic GVHD was also higher or comparable than that in the high-dose PTCy plus ATG protocols reported to be 0–18.8% with smaller study population or shorter follow-up [12–14, 17]. MAC and predominantly female donors especially female-to-male pairs [3] in the current study may be the attributable reasons as compared with previous reports on high-dose PTCy plus low-dose ATG regimens [12–14]. More recently, Wachsmuth et al. [29] showed that the dose of PTCy is important, with tested doses between 10 and 50 mg/kg/day at days + 3 and + 4 effectively prevented fatal GVHD and 25 mg/kg/day being the optimal dose in their model while doses outside this range (≤ 5 or ≥ 100 mg/kg/day) proved ineffective in preventing mortality. Overall, our current results verify the feasibility of low-dose PTCy adding to the backbone of ATG/GCSF-based regimen, and this combination for GVHD prevention may represent a promising strategy.

Understanding the mechanism of low-dose PTCy in combination with ATG is vital to explore its maneuver in innovative procedure. In our previous reported prospective trial, data suggested that low-dose PTCy is sufficient to reduce acute GVHD in mouse model, partly due to the enhancement of quick Treg reconstitution. Besides, low-dose PTCy could promote the protective effect of ATG/G-CSF on GVHD both in mouse and human sets [18]. Additionally, the timing and sequential order of given drugs may be crucial to the synergistic effect. It is conceivable that in spite of slow donor T cell depletion by ATG, donor T cells escape blockade leading to aGVHD occurrence. These escaped donor T cells exponentially expand to give rise to aGVHD when exposed to recipient antigens. Wachsmuth et al. [29] proved that PTCy induced alloreactive T cell dysfunction and suppression and supported the increasingly important role of Tregs over time post-HCT in the amelioration of chronic GVHD. Nonetheless, centers using PBSC in haploidentical transplants with PTCy have shown rates of extensive chronic GVHD as high as 38% [30]. Even with the addition of 4.5–5 mg/kg ATG to a backbone of high-dose PTCy, the rate of moderate to severe chronic GVHD was reported to be 10–13% with 2-day PTCy [13, 14] or 18.8% with 1-day PTCy [17]. This may partly explain why only total cGVHD was reduced with the current combined treatment while moderate to severe cGVHD was not. Future randomized studies including data on immune reconstitution and Treg levels will aid to further elucidate the biological aspects.

In the previous report on the combination of ATG and high-dose PTCy, the primary rejection rate was 8% [11]. In the current study, although the myeloid engraftment was 100%, hematopoietic recovery using low-dose PTCy along with ATG was slower than that in the control group. It may partly due to the immediate allo-response in which alloreactive T cells were indicated to dominate early after PTCy. It was documented that the inclusion of ATG in conditioning regimens yields quicker achievement of donor chimerism [31], and ATG was suggested to be capable to aid reliable engraftment by decreasing the aforementioned immediate allo-response early after PTCy [29]. As for disease control, it is speculated that low-dose PTCy had little impact on relapse, which is evidenced by our current data. Previous studies also demonstrated that high-dose PTCy with ATG is effective in alleviating GVHD without increasing relapse [13, 14] with the relapse incidences reported to be 16–37% [11–14, 17].

It was taken in the context of the fact that the combination of ATG and PTCy appears to increase infectious complications particularly regarding herpes virus control and may lose some of the protective effects of PTCy alone. Higher rate of CMV reactivation was noted in the combined treatment cohort as compared to the control cohort although the rate was similar to the previous report on the joint use of high-dose PTCy and ATG (74%). As compared with the control cohort, T cell impairment between donor and recipient secondary to higher extent of HLA mismatch and the dual T cell depletion with PTCy and ATG may contribute to the higher incidence of CMV reactivation. Nonetheless, only one CMV-related death occurred (Table 3). We have implemented a preemptive CMV-CTL strategy with the aim to alleviate the CMV infection risk. As for the observation that low-dose PTCy increased the risk of CMV reactivation but not the risk of relapse, although it looks in contrast to the hypothesis that PTCy acts partly by eliminating activated alloreactive T cells, it may be partly explained by recent report that CMV reactivation and expansion of CD56^bright^CD16^dim/−^DNAM1+ natural killer cells are associated with antileukemia effect after haploidentical HCT in acute leukemia [32, 33]. In addition, Wachsmuth et al. [29] revealed that 25 mg/kg PTCy did not significantly reduce CD8+ T cell proliferation while the T cell reconstitution after CMV reactivation under the combined treatment strategy needs to be elucidated. One of the characteristics of the PTCy studies has been a reduced incidence or even absence of PTLD [34]. Though we found EBV reactivation incidence was 21%, it was remarkably less in comparison with other studies using high-dose PTCy and ATG (32–64%) [11–14, 17]. In order to reduce the infectious complications, the next step with testing ATG/PTCy (low-dose) strategy may be decreasing the ATG dose also.

The limitation of the study is the non-randomized feature. We did matched-pair analysis to balance characteristics of the two populations, and the matched-pair analysis confirmed the main outcome results that we found in the standard analysis (data not shown due to the small population). Another limitation is that the comparison here is across institutions. Although the identical ATG/GCSF-based “Beijing protocol” is adopted in all the three centers (the largest and the most experienced transplant centers in China as described in the “Patients and methods” section), one cannot completely rule out that there exist small differences of medical practices between participating centers which may partly contribute to the transplant outcomes. Nevertheless, since we extended the prospective trial with the combined treatment for all HCT from MDs or CRDs in Peking University, the contemporaneous external control also transplanted from MDs or CRDs can only come from other centers. Multi-center randomized trials in different transplant settings and dose-finding studies including decreasing the ATG dose are needed to extend the practical use of the new combined treatment strategy.

In conclusion, our results showed that T cell-replete haplo-HCT after ATG/GCSF-based intensified conditioning combined with low-dose PTCy is a feasible and effective protocol, especially for patients at particular high GVHD risk. This strategy produced reliable donor cell engraftment with low rates of GVHD while keeping disease control.

## Data Availability

The datasets used and/or analyzed during the current study are available from the corresponding author on reasonable request.

## Abbreviations

aGVHD: Acute graft-versus-host disease
AL: Acute leukemia
ATG: Anti-thymocyte globulin
BU: Busulfan
cGVHD: Chronic graft-versus-host disease
CML: Chronic myeloid leukemia
CMV: Cytomegalovirus
CRD: Collateral relative donor
CSA: Cyclosporine
DFS: Disease-free survival
EBV: Epstein-Barr virus
GCSF: Granulocyte colony-stimulating factor
GRFS: GVHD-free, relapse-free survival
GVHD: Graft-versus-host disease
haplo-HCT: Haploidentical donor hematopoietic cell transplant
MAC: Myeloablative conditioning
MD: Mother donor
MDS: Myelodysplastic syndrome
MMF: Mycophenolate mofetil
MTX: Methotrexate
NRM: Non-relapse mortality
OS: Overall survival
PTCy: Post-transplant cyclophosphamide
TCR: T cell-replete
Treg: Regulatory T cell
URD: Unrelated donor
